# Management and Clinical Outcome of Penetrating Keratoplasty for Long-Term Corneal Changes in Sympathetic Ophthalmia

**DOI:** 10.1155/2011/439025

**Published:** 2011-05-05

**Authors:** Saraswathi Ramamurthi, Ebube E. Obi, Gordon N. Dutton, Kanna Ramaesh

**Affiliations:** Department of Ophthalmology, Tennent Institute of Ophthalmology, 1053 Great Western Road, Glasgow G12 0YN, UK

## Abstract

*Purpose*. To report the visual outcome of penetrating keratoplasty performed on the sympathizing eye in three cases of sympathetic ophthalmitis. *Methods*. Interventional case series of three patients, diagnosed with sympathetic ophthalmitis, with corneal changes in the form of band keratopathy and decompensation underwent penetrating keratoplasty to the sympathizing eye. They had each sustained penetrating trauma as a child and had undergone previous cataract surgery and superficial keratectomy. Two patients had undergone lamellar keratoplasty prior to this procedure. One patient had undergone trabeculectomy for glaucoma, and she was on antiglaucoma medication. The preoperative visual acuity was 1/60 in the affected eye of each patient. Penetrating keratoplasty was performed in the sympathizing eye and the donor graft size was 7.50 mm, and the host graft size was 7.25 mm. Our patients were immunosuppressed prior to the procedure to help prevent graft rejection. *Result*. At one year follow-up, a BCVA of 6/36 or better was achieved in all three patients. Postoperative examination of the fundus showed peripheral chorioretinal atrophy with pigmentary changes at the macula, accounting for the limited vision. The grafts remain clear to date, and there has been no recurrence of uveitis or rejection. *Conclusion*. Penetrating keratoplasty can be considered as a surgical option to restore useful vision in a stable sympathizing eye in sympathetic ophthalmitis, and this depends on the extent of the pathology. However, these cases require treatment with immunosuppressives to prevent graft rejection and to prolong graft survival.

## 1. Introduction

Sympathetic ophthalmia (SO) is a rare, bilateral granulomatous inflammatory condition that follows penetrating ocular injury or surgery. The sympathizing eye develops severe inflammation, and this may be worse than the inflammation in the exciting eye [1]. The course of the disease is often progressive and some of the late anterior segment manifestations such as cataract formation and corneal changes may be due to reactive and degenerative changes. Apart from cataract extraction, the surgical outcome in SO, of other types of anterior segment surgery have seldom been reported [2, 3]. We have managed three patients with long-term corneal changes that required penetrating keratoplasty to improve vision. To our knowledge, such surgical intervention to restore vision has not hitherto been reported. The aim of this paper is to report the clinical course and the outcome of the corneal grafts of these patients with SO.

## 2. Case Reports

### 2.1. Case  1

A 68-year-old female patient was referred to the corneal service with a decompensated and opaque right cornea with a vision of counting fingers. She had sustained penetrating trauma to her left eye with scissors at six years of age, and this eye was enucleated a few months later. She developed recurrent inflammation of the right eye, which was treated with oral steroids. At the age of 30 years the vision declined due to cataract formation, which was treated by intracapsular cataract extraction the following year.

**Figure 1:**
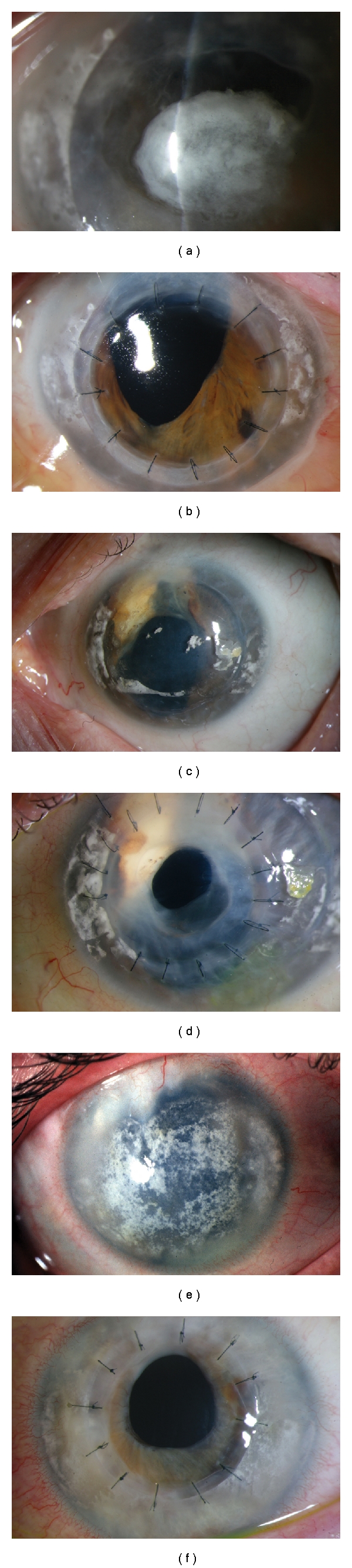
(a) Slit lamp biomicroscopy showing corneal decompensation and band keratopathy in patient 1. (b) Postoperative picture showing clear corneal graft of the same patient. (c) Preoperative slit lamp biomicroscopy showing corneal degeneration in patient 2. (d) Slit lamp biomicroscopy showing clear graft of patient 2. (e) Preoperative slit lamp biomicroscopy showing corneal changes in the form of degeneration in patient 3. (f) Postoperative clear corneal graft in patient 3.

**Figure 2:**
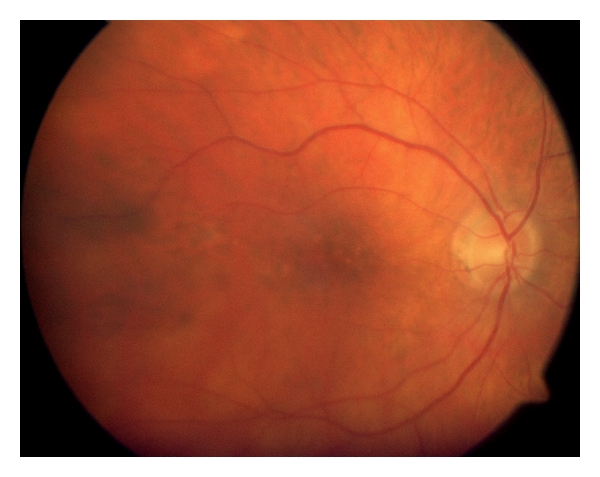
Fundus photograph showing macular scarring and peripheral chorioretinal changes.This was seen in all the three cases.

Prior to this she had undergone band keratopathy removal with EDTA on a few occasions, as well as one superficial keratectomy and one lamellar keratoplasty (8.5 mm) (Table 1). Following this procedure her low-grade uveitis recurred and the cornea developed bullous oedema, and deposition of patchy band keratopathy. The BCVA was CF in her only eye. Slit lamp biomicroscopy showed corneal decompensation and degenerative changes in the form of band keratopathy (Figure 1(a)). There was no fundus view. Ocular ultrasonography showed no evidence of retinal detachment. The patient was counselled for the high risk of graft failure and the potential risks of immunosuppressive therapy. Mycophenolate, 1 g twice a day was prescribed one month prior to the procedure. A penetrating keratoplaty was performed under general anaesthesia. The size of the donor graft was 7.50 mm (0.25 mm bigger than the host trephine). No postoperative complications were encountered during the follow-up period. Her Postoperative immunosuppressive regime consisted of MMF 1 gram twice a day with a reducing dose of oral prednisolone. The initial dose of prednisolone was 40 mg per day that was reduced by 5 mg for each week, until a 5 mg daily maintenance dose was achieved. Mycophenolate was stopped at eight months Postoperatively due to sterile pyuria, and she is currently on prednisolone 5 mg daily and topical steroids twice a day. At one year follow-up period, her BCVA was 6/18 and the graft remains clear to date (Figure 1(b)). Postoperatively the fundus examination showed a pale optic disc and chorioretinal scarring in the retinal periphery and pigmentary changes at the macula (Figure 2).

### 2.2. Case  2

A 74-year-old female was referred to the corneal services for consideration of a penetrating keratoplasty in her only eye. She had sustained injury to her right eye at the age of 3-years. She developed sympathetic ophthalmitis and the eye was enucleated at 24 years of age. She had suffered flare ups of inflammation on and off and at the age of 52 underwent intracapsular cataract extraction, followed by trabeculectomy in the same year. She developed band keratopathy, for which she underwent lamellar keratoplasty twice in 1993 and in the year 2000 (Table 1). She was on oral prednisolone 5 mg daily and topical brinzolamide. Her visual acuity was 1/60 in her left eye, the cornea was hazy due to degenerative changes (Figure 1(c)). Following detailed counselling she underwent penetrating keratoplasty. The host trephine was 7.25 mm and the donor was 7.50 mm. Postoperatively, apart from topical steroids and topical antibiotics, she was on oral prednisolone 10 mg, and she continued her antiglaucoma medication. 

At one year follow-up her best corrected visual acuity was 6/36 and she is on 5 mg daily of oral prednisolone. There were no Postoperative complications during this period and the graft remains clear to date (Figure 1(d)). Fundus examination showed pallor of the disc and peripheral chorio -retinal scarring (Figure 2).

### 2.3. Case  3

A 27-year-old male sustained penetrating trauma to his right eye as a child at 7 years of age, but unlike the other two cases had undergone primary repair. He developed sympathetic ophthalmia a few years later, and this was managed with oral steroids. He had also undergone cataract extraction to both eyes and used contact lenses to correct his aphakia. Like our other two cases, he too had undergone superficial keratectomy for band keratopathy in the sympathising eye. He was then offered and underwent a corneal graft, to the exciting right eye. This was performed under immunosuppressive cover of mycophenolate 1 g twice a day along with 40 mg of oral steroids which was then reduced to the dose 500 mg once a day and oral prednisolone 5 mg once a day. He developed glaucoma in his right eye, one year from the time of the graft and is on topical with brinzolamide and latanoprost. On examination, one year from the time of right corneal graft procedure, his BCVA was 3/60 in his right eye and 1/60 in his left eye. Anterior segment examination showed a clear right corneal graft and corneal degenerative changes in the form of band keratopathy and corneal decompensation in his left eye (Figure 1(e)). Ultrasound examination showed the retina to be attached and a single flash ERG showed a normal retinal response in his left eye. Penetrating keratoplasty was performed on the sympathizing eye, with the donor graft size of 7.50 mm and the host was 7.25 mm. Postoperatively the mycophenolate was increased to 1 g twice a day and was also on 30 mg oral prednisolone which was then tapered by 5 mg per week, and he is on the maintainance dose of 5 mg. He was also on topical steroids and antibiotics. At 10 months follow-up his BCVA was 6/36 and his graft was clear (Figure 1(e)). Fundus examination showed peripheral chorioretinal atrophy with scarring at the macula (Figure 2).

## 3. Discussion

Long-term changes of SO affect both the anterior and posterior segment of the eye. Immunohistopathological findings suggest that delayed hypersensitivity, mediated by T cells, is involved in the pathogenesis of SO [4, 5]. Recurrences and low-grade inflammation are common. The pathological changes observed in long-term SO are aggravated by reactive and degenerative changes secondary to chronic inflammation specifically in the anterior segment of the eye. The principle posterior segment changes comprise chorioretinal degeneration, macular degeneration, and optic atrophy. On the long term, the anterior segment changes include band keratopathy, development of cataract, corneal oedema/bullous keratopathy, endothelial loss, iris atrophy, and ciliary body atrophy and traction. The mechanisms by which chronic inflammation damages the cornea, lens, retina, and uveal tissue are complex and may be due to the sustained and direct cytopathic action of inflammatory mediators, free radicals, and proinflammatory cytokines [2, 6]. Further chronic use of oral steroids may contribute to cataract development and cataract surgery may aggravate the depletion of endothelial cell loss. 

All three patients who were managed with penetrating keratoplasty had several high-risk factors for graft failure. Chronic uveitis and the presence of anterior synechiae are known inflammatory status, antigen presenting cells that may facilitate recognition of foreign antigens and increase the chances of graft rejection are primed. Further, the risk of graft failure was high in these patients as they all undergone previous intraocular surgery, partial thickness corneal grafts, and raised intraocular pressure all of which are all known to increase graft failure rates [7–11].

All our patients had chronic inflammation for number of years but were quiescent for at least six months preceding surgery. Intraocular surgery can reactivate Intraocular inflammation in eyes that had multiple episodes of chronic uveitis [12], and this can impair graft survival. Although penetrating keratoplasty in the eye with chronic uveitis or SO has not been reported, cataract surgery in the sympathizing and the exciting eye have been performed with good visual outcome [2, 3], and these patients need additional anti-inflammatory cover to control inflammation. 

Our patients carried a high risk of rejection but also reactivation of Intraocular inflammation. The immune mechanisms of graft rejection in SO are T-cell-mediated delayed hypersensitivity reactions. The initial response after antigen presentation in the case of endothelial surface antigens is T-cell activation. This results in activation of the proinflammatory cascade and destruction of endothelium, mediated by the killer cells and other cytokine-mediated cytotoxicity. Once the endothelium is completely destroyed this antigen-driven response abates, and there is no further inflammation. However in the case of SO initial antigen-driven activation of T cells may follow a chronic course. During the chronic phase, that is nonantigen driven, nonspecific T-cell activation, and activation of macrophages and other myeloid cells can give rise to another phase of inflammation and tissue damage. Therefore in our patients the therapeutic target was to control antigen-driven T-cell response (related to endothelial antigen and possibly antigens related to SO) and nonspecific (related to chronic phase of SO) T-cell and macrophage activation, inflammation and tissue damage. During the immediate Postoperative period the factor of surgical trauma-induced inflammation needs to be taken into consideration. 

To improve the chances of graft survival and to prevent recurrence of Intraocular inflammation, T-cell modulator is considered. Calcineurin inhibitors such as cyclosporine or tacrolimus or mycophenolate mofetil (MMF) that can modulate T-cell function and may control chronic uveitis components and also improve graft survival. 

Both calcineurin inhibitors cyclosporine [13, 14] and tacrolimus have proven beneficial in controlling uveitis [15, 16] and increasing graft survival [17, 18]. However the bioavailability of cyclosporine is highly variable and difficult to predict [19–22]. The pharmacokinetic properties of cyclosporine can be significantly affected by age [19, 20], ethnicity [21, 22], variability of gastrointestinal milieu, concomitant ingestion of certain foods [23] bile flow and medications [24], and coexisting pathology such as diabetes [22]. Similarly the absorption of tacrolimus is also highly variable leading to varying blood concentration profiles and peak blood or plasma concentrations being reached in 0.5 to 6 hours [25]. However bioavailability can vary from 5% to 67% with a mean of 29% [26].

 The pharmacokinetics of cyclosporine and tacrolimus are complex and unpredictable with a narrow therapeutic index unique to each patient, as well as variable absorption, distribution and elimination making therapeutic drug monitoring important [27]. 

At the time when our patients underwent surgery, MMF had been reported as an effective agent in both controlling uveitis [28] and graft rejection [17]. MMF has been successfully used in the management of organ transplantation and chronic uveitis with fewer side effects [28, 29]. For these reasons we elected to use MMF as an immunosuppressive agent for two of our patients. Patient number two had cardiomyopathy and was a generally frail patient and her cardiologist advised against immunosuppressive medications. Hence she was on 10 mg of oral steroids. Patient number three was already on mycophenolate from the time of his initial graft procedure to the other eye. Mycophenolate was stopped eight months Postoperatively in patient one, due to sterile pyuria. None of our patients developed any flare-up of uveitis or graft rejection during the follow-up period, and the grafts remain clear to date.

In future, biological agents may be considered to aid in managing similar situations but currently the evidence for the use is sporadic. Infliximab has been reported to be effective in controlling chronic uveitis and Behcet's disease [30–32]. Although infliximab has been successfully used in the management of intestinal transplant rejection [33–35], it has not been used in the management of corneal graft rejection and the high incidence of side effects is a concern [36]. There are only a few isolated case reports regarding the beneficial use of biological agents for graft rejection [37].

To conclude, penetrating keratoplasty can be considered a safe surgical option to restore useful vision in a stable sympathising eye. Preoperative and Postoperative control of inflammation with immunosuppressive agents is imperative to prevent rejection in these high-risk cases.

## Figures and Tables

**Table 1:** This table shows the list of procedures before PK in the sympathising eye.

	Surgery 1	Surgery 2	Surgery 3	Surgery 4
Case 1	Cataract surgery	Superficial keratectomy	Lamellar keratoplasty	Penetrating keratoplasty
Case 2	ICCE with trabeculectomy	Lamellar keratoplasty	Superficial keratectomy	Penetrating keratoplasty
Case 3	Cataract surgery	Superficial keratectomy	Penetrating keratoplasty	
